# Talking but not always understanding: couple communication about infertility concerns after cancer

**DOI:** 10.1186/s12889-021-10188-y

**Published:** 2021-01-19

**Authors:** Alexandra Hawkey, Jane M. Ussher, Janette Perz, Chloe Parton

**Affiliations:** Translational Health Research Institute, School of Medicine, Western Sydney University, Locked Bag 1797, Penrith South, 2751 Australia

**Keywords:** Cancer, Cancer survivorship, Infertility, Relationships, Couple communication, Oncofertility, Fertility

## Abstract

**Background:**

Cancer related infertility can have an impact on couple relationships, with evidence that couple communication facilitates coping. However, little is known about the ways in which couples communicate about cancer-related fertility concerns. The aim of this article is to examine couple communication about fertility concerns in the context of cancer, and the perceived quality of such communication from the perspective of cancer survivors and their partners.

**Methods:**

Eight-hundred and seventy-eight cancer survivors (693 women, 185 men) and 144 partners (82 women, 62 men), across a range of tumour types and age groups, completed a survey which examined cancer related fertility concerns. Seventy-eight survivors (61 women and 17 men), and 26 partners (13 women and 13 men), participated in semi-structured interviews, in order to examine the subjective experience of fertility concerns in-depth. Thematic analysis was used to analyse the interviews and open ended survey questions. Valid percentages for single items from the relationships subscale of the Fertility Preservation Inventory (FPI) related to qualitative themes, identified frequency of responses.

**Results:**

The major theme was ‘talking but not always understanding”. 89.6% of cancer survivors and partners (95.1%) reported working well together handling fertility questions (FPI), but agreed that communication could be improved (65.9% survivors; 65% partners). Open and honest couple communication was associated with feelings of support, understanding and relationship growth, including perception of partner comfort (79.2% survivors, 81.6% partners). However, 32% survivors and 31.1% partners concealed fertility concerns to avoid upsetting their partner, or reported that their partner doesn’t understand their fertility concerns (survivors 25.5%, partners 14.6%), with 14.1% of cancer survivors and 19.4% partners reporting fear of relationship breakdown because of fertility issues. Fear of rejection when forming new relationships, and concerns about how to talk to future partners, was reported by non-partnered individuals.

**Conclusion:**

Health-care professionals should include partners of cancer survivors in fertility discussions. Couple interventions developed in general psycho-oncology should be extended to the domain of fertility, in order to facilitate effective couple communication. Communication in future relationships needs to be addressed for single people and adolescents and young adults (AYAs) who have fertility concerns.

**Supplementary Information:**

The online version contains supplementary material available at 10.1186/s12889-021-10188-y.

## Background

There is increasing recognition of the influence of the couple relationship on the experience of cancer survivorship [1] with cancer described as a ‘we-disease’ [2] and cancer coping a dyadic process [3]. Levels of distress of cancer survivors [4] and their familial carers [5] are higher than population norms, with significant correlations found between distress of cancer patients and their intimate partners [6]. At the same time, a positive couple relationship context and partner support is associated with adjustment and coping following cancer diagnosis [7, 8]. This has led to the development of couple based psychosocial interventions to improve coping and psychological wellbeing in the context of cancer [9–12]. Many of these interventions include a focus on improving couple communication, which is acknowledged as central to relationship coping [13–15].

It has been demonstrated that couples who address cancer related problems or concerns with mutual discussion, open expression of feelings, empathy, and joint resolution of problems, are more able to engage in effective emotion and problem focused coping [16, 17]. Adopting these strategies is associated with lower levels of distress, and higher levels of relationship satisfaction [18–20], and can lead to couples finding benefits in the cancer experience, such as personal growth and relationship closeness [2, 21]. Conversely, many partners are over-protective towards the cancer survivor, engaging in what has been described as “protective buffering” in an attempt to prevent distress [22, 23], “disengaged avoidance” [2] involving complete denial of cancer or its effects, or “holding back” [20] and “self-silencing” their own needs and concerns [24, 25]. Whilst these communication practices may appear to be functional in protecting against distress, they are associated with poorer mental health in both patients and their partners, as well as lower relationship satisfaction [1, 18–20, 26], as they obviate engaged support [2], and can alienate the patient [23]. In combination, these findings across research studies demonstrate that cancer survivorship is an experience that should be examined from a relationship perspective, including a focus on couple communication and coping [27].

Impaired fertility is estimated to affect between 25 and 60% of people with cancer [28, 29], caused by the disease itself, or resulting from gonadal damage following chemo-therapy, radio therapy, or bone marrow transplantation [28, 30]. There is growing evidence that concerns about infertility can be the most difficult long term effect of cancer treatment [31], associated with psychological distress, poor quality of life, low self-esteem, and changes to body image and gender identity [32–35]. Many women and men cancer survivors report a desire for parenthood [36–38], with fertility associated with feeling normal [39], and parenthood acting to “close the door” on cancer ([40] , p.105). However, rates of parenthood among cancer survivors is lower than in the general population [41], particularly for women [42, 43]. This has led to calls for further research on the psychological consequences of fertility concerns post-cancer [28, 34, 40, 44, 45].

Infertility is known to have an impact upon couple relationships within the general population [46, 47]. Couples who experience compatibility and congruence in coping with infertility have been reported to experience better communication quality, with positive outcomes for mental health and relationship outcomes [47]. In the context of cancer, infertility distress has been found to be associated with relationship dissatisfaction [32, 48], fears of abandonment or rejection by current or future partners [38, 49], and reduced sexual satisfaction [33]. However, many couples adopt strategies to facilitate coping, including acceptance of infertility and privileging of survival, focusing on relationship growth, optimism, and nurturing in other ways [48]. Couple communication has been extensively examined in the context of sexual difficulties following cancer [50, 51], and reported to be a predictor of sexual and psychological wellbeing [52], and renegotiation of sexual activities [53]. However, there is an absence of research examining the nature and quality of couple communication about fertility concerns after cancer. This has led to a plea for research in this area, to examine the “specific communication challenges couples encounter when discussing oncofertility concerns” ([54] , p. 75).

It has been reported that women with breast cancer describe their intimate partner as the most helpful person in discussions about infertility [55], however little is known about the nature or experience of such couple discussions [56]. Research on cancer related fertility communication has focussed on healthcare professional - patient communication [43], from the perspective of both clinicians [57] and patients [58]. This reflects recognition that such discussions are “a crucial aspect of high quality healthcare” which assists patient adjustment [59]. Indeed, there have been calls to incorporate partners and spouses into discussions with health care professionals about fertility concerns after cancer [54]. Understanding how couples communicate about fertility issues, and the perceived quality of such communication, is an essential step in the effective incorporation of partners into these discussions. The aim of this article is to address this gap in the research literature, though examining couple communication about fertility concerns in the context of cancer, and the perceived quality of such communication, from the perspective of cancer survivors and their partners.

Previous research on fertility concerns in the context of cancer has been criticised for being small scale, focusing on cancer that affects the reproductive organs, and recently diagnosed young women, with patients recruited from a single clinical site [37, 60]. There is evidence that a wide range of cancers and cancer treatments may impact upon fertility [34, 61], and fertility related distress occurs across tumour type [32, 33]. This suggests a need for inclusion of a broad range of cancer types in research on the experience of fertility concerns following cancer. Previous research has also focused on adolescents and young adults (AYAs) [37, 60, 62], reinforced by fertility guidelines which focus on AYAs [63, 64]. This can result in the fertility concerns and experiences of older cancer survivors being overlooked [42]: in Australia those over the age of 25 [65], in the USA those over age 39 [66], reflecting different age ranges in the definition of AYA cancer survivor. Equally, the majority of research on cancer and fertility has focused on the cancer patient or survivor, with minimal attention paid to the experience of partners [29, 43], despite increasing recognition of the psychosocial needs of partners [67, 68].

The aim of the present study was to address identified gaps in the research literature through investigating the following research questions in women and men cancer survivors and partners, across a range of cancer types and age groups: How do couples communicate about cancer related fertility concerns? What is the perceived quality of such communication from the perspective of cancer survivors and their partners?

## Method

### Design

This study was part of a broader program of mixed method research examining the experience and impact of cancer related fertility concerns from the perspective of cancer survivors, their partners, and health care professionals [32, 38, 48, 57, 58, 69, 70]. The present article focuses on the nature and quality of couple communication about cancer related fertility concerns, drawing on closed and open ended survey items completed by a broad cross section of women and men cancer survivors, across tumour types and age groups, and in-depth one-to-one interviews conducted with a purposively selected subsample of survey respondents.

### Participants and procedure

Participants opted-in to take part in the study, in response to information about the study circulated across Australia through cancer support groups, social media, media stories in local press, advertisements in cancer and carer-specific newsletters, hospital clinics, and local Cancer Council Websites and telephone helplines. Participants completed an online or postal survey examining their experiences of fertility concerns post-cancer. At the end of the survey, participants indicated whether they would like to be considered to take part in an interview, to discuss fertility concerns in more depth. Purposive sampling [71] was used to select interview participants, with the aim of gaining insight into the experience of people across of variety of age groups and cancer types.

### Interview and survey

Participants were provided with an information sheet describing the purpose of the interviews, and completed a written consent form prior to the interview. Interviews were conducted one-to-one by telephone, taking approximately 1 h, and were digitally recorded and transcribed verbatim. The interviews were conversational in style, with the wording and formatting of questions used flexibly to suit the particular context of the participant [72]. The interview questions covered experiences of cancer related infertility, communicating with fertility concerns with partners and healthcare professionals, and support needs (Supplementary file 1). All the interviews were transcribed verbatim by professional transcribers, and integrity checked for accuracy by a member of the research team.

The survey included a series of closed and open ended questions about fertility and cancer (Supplementary file 2). In this paper, we focus on participant responses to a series of items that related to themes identified in the qualitative data, in order to obtain an indication of the frequency of responses across gender, for cancer survivors and partners. This included single items drawn from the relationships subscale of the Fertility Problems Inventory (FPI) [73] - a validated measure which assesses infertility-related stress dimensions, using a six-point Likert scale. These items were: “My partner and I work well together handling questions about our fertility”; “when I talk about fertility issues, my partner seems comforted by my comments”; because of infertility, I worry that my partner and I are drifting apart”; “I can imagine separating because of fertility issues”; “when we try to talk about fertility issues, it seems to lead to an argument”; “I can’t show my partner how I feel [about infertility] because it will make him/her feel upset”; “my partner doesn’t understand the way fertility issues affect me”; “it bothers me that my partner reacts differently to our fertility issues”; “my partner and I *could* talk more openly with each other about our fertility issues”. We also used open ended items devised specifically for this study, examining couple communication about fertility concerns: Have fertility issues affected your relationship(s) or your ability to form a new relationship? (please explain). Please explain what was satisfying, or dissatisfying, about discussing fertility issues with your partner, family or friends.

### Analysis

Thematic analysis [74] was utilised for the qualitative survey responses and interviews. The analysis was conducted using an inductive approach, meaning that the development of themes was data driven, rather than being based on pre-existing research on couple communication about cancer related fertility concerns. This process involved researchers reading through the responses to each interview in order to identify first order codes such as ‘disclosure of fertility concerns’, ‘benefit finding’, ‘relationship changes’ and ‘relationship support’. Broader team members brought suggestion of the first order codes to the meeting, and the final coding frame was devised through a process of consensus. The entire dataset was then coded using NVivo, a computer package that facilitates organisation of coded qualitative data. The senior member of the team (JU) checked the coding to ensure consistency across codes and coders. All of the coded data was then read through by two members of the team, and summaries of the themes within the coded data produced. Codes were then grouped into higher order themes. This process involved checking for emerging patterns, variability and consistency, commonality across participants, and for uniqueness within cases, in order to identify the experiences of cancer related infertility concerns. Through this process, a final overarching theme related to couple communication was developed from the interviews and open ended survey responses, as well as a number of sub-themes. In the presentation of data below, participants are identified as a cancer survivor or as a partner, along with their type of cancer, and age range, rather than actual age, to maintain anonymity. Pseudonyms have been allocated to interview participants, and gender indicated for open ended survey respondents.

Descriptive statistics were used to calculate valid percentages for items on the FPI separately for cancer survivors and partner samples. Fishers exact test was used to examine differences between women and men per item, separately for cancer survivors and partners.

## Results

### Participants

Eight hundred and seventy-eight people cancer survivors (693 women, 185 men) and 144 partners (82 women, 62 men) completed the survey. The sample size was determined by the number of responses, as it was an opt in survey. The average age of cancer survivors was 42.53 years (SD = 14.21), partners 44.9 (SD = 13.75), and average time from diagnosis 6.22 years (SD = 7.01) for cancer survivors and 5.90 years (SD = 5.96) for partners. The sample was drawn across cancer types including breast (cancer survivors 45.2%, partners 29.4%), hematologic (cancer survivors 14.9%, partners 18.4%), gynaecological (cancer survivors 10.4%, partners 3.7%), genitourinary (cancer survivors 11.3%, partners 25%), gastrointestinal (cancer survivors 4.6%, partners 9.6%), neurologic (cancer survivors 4.0%, partners 5.1%), head and neck (cancer survivors 3.3%, partners 2.2%), skin (cancer survivors 2.7%, partners 2.9%), musculoskeletal (cancer survivors and partners 2.9%), and respiratory (cancer survivors and partners 0.7%). The sample was almost exclusively heterosexual (98%), with 71% PWC and 100% partners reporting that they were currently in a relationship. Full demographic details are presented in Table 1.

**Table 1:** Sociodemographic and cancer characteristics by gender

	Women		Men	
	***n***	***%***	***n***	***%***
Age (in years)	42.58	14.21	50.66	20.61
Years since first diagnosis	5.95	7.13	7.26	6.81
Ethnicity				
Anglo-Australian	573	89.7	156	87.6
Asian^a^	19	3.0	4	2.2
Other^b^	27	4.2	7	3.9
Missing	20	3.1	11	6.2
Education level				
Still in school	26	3.7	3	1.6
Completed Secondary School	151	21.7	58	31.4
Trade Certificate or Diploma	188	27.1	58	31.4
Tertiary Degree or Higher	330	47.5	66	35.7
Employment status				
In paid work	419	60.2	77	41.2
Not in paid work	123	17.7	28	15.0
Other^§^	154	22.1	82	43.9
Relationship status				
Partnered	498	71.4	135	72.6
Not partnered	199	28.6	51	27.4
Sexual identity				
Heterosexual	679	98.0	173	94.0
Non-heterosexual	14	2.0	11	6.0
Parenthood status (parity)				
Nulliparous	302	43.3	68	36.4
Parous	396	56.7	119	63.6
Stage of cancer at diagnosis				
Early	457	67.4	90	51.4
Advanced	120	17.7	55	31.4
Unknown	101	14.9	30	17.1
Cancer site				
Breast	390	56.8	2	1.1
Hematologic/Blood	88	12.8	41	22.8
Gynaecologic	90	13.1	–	–
Genitourinary	7	1.0	91	50.6
Musculoskeletal	16	2.3	9	5.0
Neurologic	22	3.2	13	7.2
Digestive/Gastrointestinal	33	4.8	7	3.9
Skin	16	2.3	7	3.9
Head and Neck	20	2.9	9	5.0
Respiratory/Thoracic	5	0.7	1	0.6
Cancer type				
Non-reproductive	200	29.1	87	48.3
Reproductive	487	70.9	93	51.7

^a^Includes South East Asia, India and Sri Lanka

^b^Other includes: Australian Aboriginal, Middle East, Northern European, Latin America, Pacific Islands

Seventy-eight cancer survivors (61 women and 17 men), and 26 partners (13 women and 13 men), who accepted the invitation to take part in interviews were interviewed. We purposively selected interviewees across gender, cancer type and stage, and age, and interviewed a larger number of women due to the wider range of experiences reported by women participants, which meant that it took longer to reach information power, the number of interview participants required to meet the aims of the study [75].

### Talking but not always understanding: couple communication about infertility concerns after cancer

**Fig. 1:**
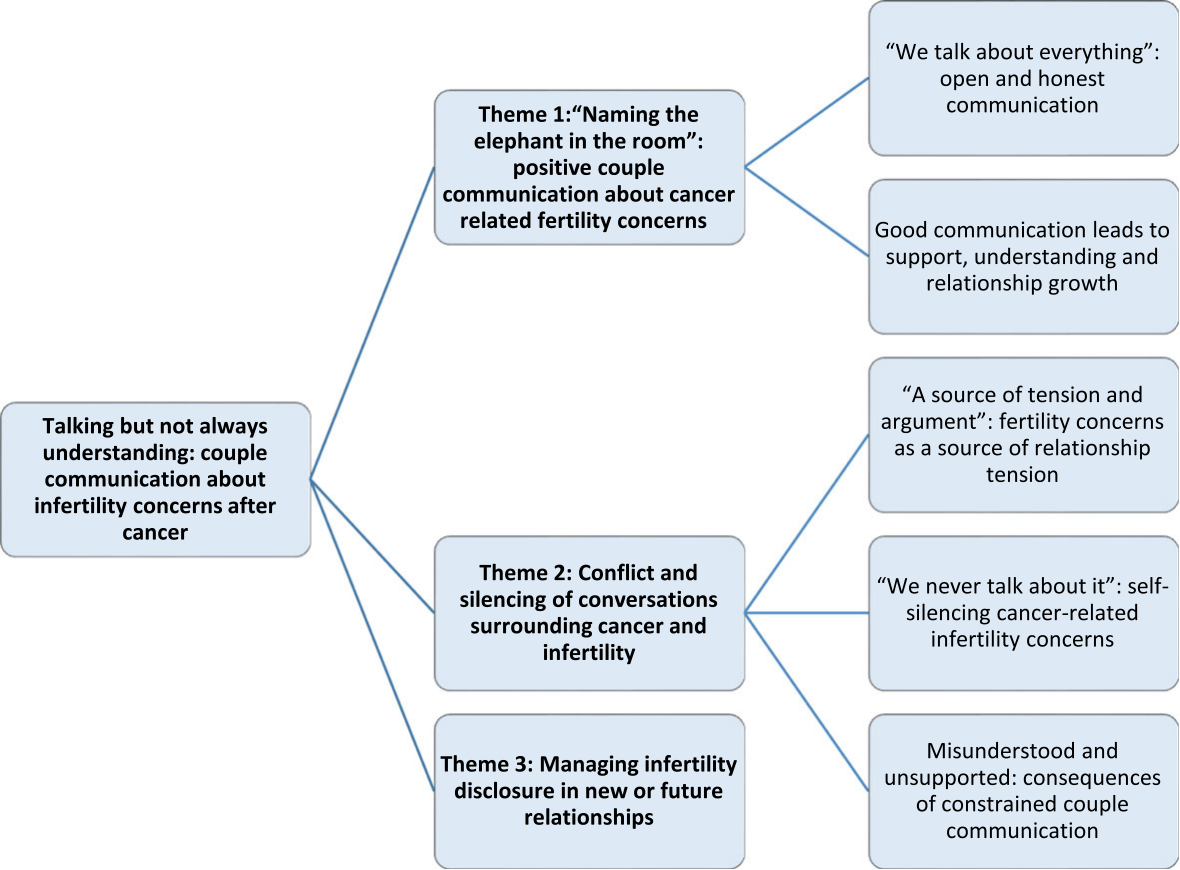
Thematic map

The final overarching theme developed from the interviews and open ended interviews was: Talking but not always understanding: couple communication about infertility concerns after cancer. There were a number of subthemes, outlined in the thematic map (Fig. 1), and described in the analysis below. FPI responses to items aligned with the subthemes are summarised for cancer survivors and partners, with frequency distribution across gender, and results of the Fisher exact test, examining significant differences across gender, are presented in Supplementary Table 3 (cancer survivors) Supplementary Table 4 (partners).

#### “Naming the elephant in the room”: positive couple communication about cancer related fertility concerns.

##### “We talk about everything”: open and honest communication

 The importance of “working together” as a couple to address cancer related fertility concerns - was acknowledged by both cancer survivors and partners. The majority of participants (survivors, 89.6%, *n* = 415; partners, 95.1%, *n* = 98) agreed with the survey item “My partner and I work well together handling questions about our fertility” (FPI). In the interviews and open ended survey responses, many participants emphasised the importance of open and honest communication with their partners, stating that they were “very open with each other” (Harry, survivor, 40–45 years, Hodgkin’s Lymphoma) and that they talk about “everything and anything” (Nina, survivor, 35–40 years, breast).

Both myself and [partner] do like to be honest and open with each other. We’ve been like that from the start, so, there was a lot of late nights lying in bed talking about it (Christine, partner, 25–30 years, haematological).

Good quality couple communication was described as something that occurred across the cancer continuum, starting “from the very beginning” (Christan, survivor, 35–40 years, testicular), “straight after he was diagnosed” (Pam, partner, 15–20 years, haematological), and “through treatment and recovering” (survivor, man, 35–40 years, testicular). It also involved a dyadic “back and forth, no argument, just discussion” (Victor, survivor, 35–40 years, leukaemia), where partners don’t “shy away” (Imogen, survivor, 40–45 years, breast) from difficult conversations. Participants also spoke about the importance of frequent, “in depth discussions” (survivor, woman, 40–45 years, breast), where couples “talk about everything” (Olivia, survivor, 20–25 years, lymphoma) in relation to their cancer and fertility experiences. Having a history within the couple relationship of effective communication was reported to have facilitated discussion of fertility issues after cancer:

We’d already talked about [fertility], before we got married even, so we were both on the same page about that. … When all the cancer happened to both of us, I found it, I found her easy to talk to about that sort of stuff. We didn’t really disagree on anything, except I felt like I needed a bit of time to recover from chemo and treatment before we moved to IVF, so, yeah, we didn’t really have any communication issues or relationship issues (Liam, survivor, 35–40 years, testicular).

Good quality communication and working together to address cancer related fertility concerns was identified as having had a number of positive impacts on couple relationships, which are outlined below.

##### Good communication leads to support, understanding and relationship growth

For the majority of participants, open and honest communication was reported to have contributed to feelings of support, empathy and understanding within the relationship. This was reflected in positive responses to the survey item “when I talk about fertility issues, my partner seems comforted by my comments” (survivor, 79.2%, *n* = 366; partner 81.6%, *n* = 84). It was also mirrored in interviews and open-ended survey questions, with Laurence (survivor, 35–40 years, testicular) telling us his partner did “everything possible to make me feel okay and make me feel good … she wasn’t just saying it to make me feel happy … she was incredibly supportive” and Georgina (survivor, 40–45 years, breast) said, “we talk about it and he’s sensitive to my feelings … it’s not something he ignores or disregards.” For some participants, talking about infertility concerns also meant that there was a process of shared-decision making and support in relation to fertility preservation and treatment options. For instance, one survey participant said the, “discussion and reviewing the decisions we had made about fertility preservation, about the changes to my body, it felt good to get support for the issues at the time” (survivor, woman, 35–40 years, breast) and Julia (survivor, 25–30 years, Hodgkin’s Lymphoma) said, “it’s been more the two of us, as opposed to just me.” Participants also described open communication about infertility as having reduced their anxiety and facilitated coping. Lara (survivor, 20–25 years, leukaemia) told us, it was “good to vent about it, rather than worrying about it on the inside” and Lily (survivor, 20–25 years, Hodgkin’s Lymphoma), said that she “definitely feel [s] better having somebody to talk about it [with].”

In many instances open and honest communication about cancer and infertility was also reported to have facilitated relationship growth. Participants told us that a cancer diagnosis and subsequent conversations about infertility “brought us closer together” (partner, woman, 30–35 years, testicular), “confirmed our relationship even more” (Christian, survivor, 35–40 years, testicular) and “only made us stronger” (Monica, survivor, 25–29 years, breast), leading some to say “our relationship is amazing now … we couldn’t be happier” (Ava, partner, 30–35 years, testicular). Specifically, couples described that discussing a difficult topic like infertility, also “taught us how to communicate” (Denise, survivor, 30–35 years, breast). For example, Sandra (survivor, 30–35 years, breast) described that;

It’s helped open up a lot deeper dialogue of communication between us … as hurtful and emotional as it is, we can sort of rationally and methodically and logically talk about something. It’s actually strengthened that aspect of our relationship, our communication, our ability to talk things through without clamming up or shutting down or someone walking out.

Open and honest communication through naming the “elephant in the room” (Polly, 30–35 years, survivor, breast) resulted in couples feeling that they had an understanding of what each person was going through emotionally. This in turn lead to greater feelings of support and comfort within the relationship, enhancing relationship satisfaction. For instance, Patricia (survivor, 30–35 years, breast) said, “we sort of comfort each other … support each other through the concerns” and a survey participant (survivor, woman, 30–35 years, breast) described, “my partner and I had several discussions about fertility issues and I feel like everything was out in the open and clearly discussed with both of us, so this was satisfying.”

#### Conflict and silencing of conversations surrounding cancer and infertility

##### “A source of tension and argument”: fertility concerns as a source of relationship tension

The negative impact of cancer related fertility concerns on relationships was reported by a minority of participants (survivors, 8.4%, *n* = 39; partners, 7.8%, *n* = 8) who agreed with the FPI items that “because of infertility, I worry that my partner and I are drifting apart”, and that they could “imagine separating because of fertility issues” (survivor, 14.1%, *n* = 65; partner, 19.4%, *n* = 20). In some instances, relationship strain as a consequence of infertility was described as a “contributing factor” (Miranda, 38 years) to the “slow and steady decay of the relationship” (Shirley, 20–25 years). For other participants, infertility following cancer was explicitly stated to be “the main reason we broke up” (survivor, woman, 45–50 years, cervical/ovarian cancer).

Conflict in communication was identified as a factor in relationship strain for some participants (survivor, 7.3%, *n* = 34; partner, 4.8%, *n* = 5) who agreed that “when we try to talk about fertility issues, it seems to lead to an argument” (FPI). Across open-ended survey responses and interviews a number of participants positioned the discussion of cancer-related infertility as a “source of tension and argument” (Survey, partner, man, 30–35 years, breast), a “cause of extra stress” (Survey, partner, man, 40–45 years, lung), something that “caused conflict” (Survey, survivor, woman, 35–40 years, breast) or a “massive fight” (Shirley, partner, 20–25 years, brain) within their intimate relationships.

For a number of younger participants, a cancer diagnosis and potential infertility issues as a consequence of treatment brought the discussion of childbearing up prematurely in relationships, which participants described as not “normal” and “unfair”. As Amanda (25–30 years, haematological) told us,

I’m a 26-year-old woman that doesn’t want to scare her boyfriend away by talking about babies now. It is quite upsetting that I have to think about these things and not just go through the normal process of the relationship and normal conversations that you’d be having. Not conversations, oh look, we might not be able to, or we might have to try alternatives ways we don’t know yet. So, that bit’s very difficult.

Similar sentiments were shared by Roxanne (20–25 years, survivor, breast) who said, “I do remember having a discussion with him early on, just saying it’s not really fair … just having to think about all of this [fertility], when we’re not ready, not at that stage … it kind of pushes your relationship forward at a really quick pace.” These accounts suggest that open discussion of fertility within couple relationships is not always a positive experience for cancer survivors and their partners, particularly if they are younger.

##### “We never talk about it”: self-silencing cancer-related infertility concerns

Absence of discussion of fertility concerns was commonly reported as a means of avoiding a distressing issue. Many participants reported hiding their feelings about infertility to protect their partner (survivor, 32%, *n* = 149; partner, 31.1%, *n* = 32) agreeing with “I can’t show my partner how I feel [about infertility] because it will make him/her feel upset” (FPI). As a consequence, participants described self-silencing their concerns surrounding infertility, stating that they “can’t sort of talk about it” (Tyrone, partner, 30–35 years, bowel), “we’ve not actually spoken about that in much detail” (Claire, survivor, 30–35 years, cervical), or that they have “never really talked about how we each felt about it” (Sylvia, survivor, 55–60 years, kidney).

For many participants, couple communication about cancer-related infertility concerns was described as “painful”, “upsetting” and “raw”. As Fiona (survivor, 35–40 years, breast) described, “it’s a painful subject, we probably don’t talk about it all that often … we both feel the other is quite sad about the issue, perhaps we don’t want to broach it.”. Brienne (survivor, 25–30 years, breast) told us, “before my diagnosis [having a child] was something we discussed pretty often. I don’t talk about it now because it upsets me so I’m trying to avoid that.” Adam (partner, 35–40 years, cervical) told us that his wife wanted to try for another child, and he didn’t, and “I guess the way we [should be] dealing with it is talking, but no, we’re not dealing with it.” Due to the distressing nature of fertility conversations, some cancer survivors spoke about ending discussions about fertility when initiated by their partners, as a means of coping. Chloe (survivor, 30–35 years, breast) told us;

I just completely broke down, I couldn’t handle it … in the end I just ended up shutting down the conversation … it’s just one of those underlying things we don’t talk about at the moment … I have just kind of had to shut down, because I can’t cope with it.

Cancer survivors also described their partners avoided conversations around infertility because they did not want to add to existing cancer related distress. Lily (survivor, 20–25 years, Hodgkin’s Lymphoma) said her partner “wouldn’t want to talk about it” because, “he knew it was going to upset me” and Francesca (survivor, 25–30 years, breast) described that it was “really hard for him to talk about because he would see me so upset...he didn’t want to pressure me.” At the same time, cancer survivors spoke about avoiding discussion of infertility as they did not want to add to the burden their partners were carrying as a carer. Hope (survivor, 20–25 years, Hodgkin’s Lymphoma) told us, “I’d never want to burden him with any of my issues or anything, because I knew it was hard enough already as it is” and Amanda (survivor, 20–25 years, Hodgkin’s Lymphoma) described, “it’s a case of yes, it’s on my mind, but I don’t want to burden him with it.” This meant that even when participants were “having an upset day” when thinking about infertility it wasn’t something they “liked to burden other people with” (Olivia, survivor, 20–25 years, Lymphoma).

A number of participants attributed the absence of discussion of fertility concerns to gender differences in communication, described by one woman as “just the typical woman/male thing” (Ava, partner, 30–35 years, testicular). Some women positioned themselves as being “over thinkers”, “worry warts” and “emotional”, while men were described as “inward”, “level-headed”, “strong” and “private”, meaning they were more reluctant to talk through fertility concerns. As Heather (survivor, 45–50 years, gynaecological) told us,

When you have the cancer, you want to be able to discuss … fears around surviving and fertility … my feeling is that a lot of the men don’t want to talk about it … that stoic upper lip kind of thing.

Similar sentiments were evident in Sophie’s (survivor, 35–40 years, leukaemia) account, when she discussed doing “most of the thinking in our relationship …. I think out loud, he does his thinking in private which doesn’t make for a very two-way conversation about extraordinarily difficult topics”. At the same time, a number of men described fertility discussion as “uncomfortable”, meaning “I haven’t really talked to anyone about it” (Kevin, partner, 25–30 years, bowel). Stuart (partner, 49–50 years, breast) acknowledged difficulties in discussing fertility with his wife, and said “I guess we really needed counselling, we had no counselling”. Absence of talk did not necessarily mean absence of support, however, as Brienne (survivor, 25–30 years, breast) said of her husband, “he usually doesn’t say a lot. I usually just get a very long hug and – and by the time that’s over with I’ve calmed myself down.” These accounts suggest that while communication about cancer and infertility may be avoided by both men and women, gendered expectations around what is considered appropriate communication within heterosexual couples may act as a further barrier to discussion of fertility concerns.

##### Misunderstood and unsupported: consequences of constrained couple communication

A lack of open communication or poor quality communication was reported to have had a number of negative consequences for couple relationships. In the survey, 25.5% (*n* = 118) of cancer survivors and 14.6% (*n* = 15) of partners agreed that “my partner doesn’t understand the way fertility issues affect me”. A number of survey participants also agreed that “it bothers me that my partner reacts differently to our fertility issues” (survivor, 18.4%, *n* = 85; partner, 26.2%, *n* = 27). Consequently, a majority of survey participants (survivor, 65.9%, *n* = 304); partner, 65%, *n* = 65) agreed with the survey item “my partner and I *could* talk more openly with each other about our fertility issues” (FPI).

In the interviews and open ended survey items, participants described poor communication as leaving them feeling unsupported and alone. Melanie (survivor, 40–45 years, colorectal) described her partner as “not very good at talking about personal stuff, he’s very superficial in conversations” and as a consequence felt like she was “not getting any support.” Similar sentiments were apparent in Abigail’s account (survivor, 30–35 years, breast,) who described her partner as “incredibly unsupportive throughout the whole process” and that she “felt really alone during that period” of addressing fertility concerns. This in turn led to relationship difficulty and dissatisfaction, as described by one survey participant, “[I’m] unsatisfied with my husband because I think he holds back about how he really feels and it has caused problems in our relationship” (survivor, woman, 35–40 years, breast cancer).

The major breakdown of our marriage was his communication or lack of communication, whereas I needed to talk about all these things, it wasn’t something that he was willing, or capable of discussing … [it] made for a horrible experience, so much harder to get through having to deal [with it] by yourself (Charlotte, survivor, 40–45 years, breast).

In combination, these accounts suggest that a lack of open communication surrounding fertility concerns can lead to couples feeling unsupported, dissatisfied with their relationship and in some cases lead to relationship breakdown.

#### Managing infertility disclosure in new or future relationships

Difficulties associated with fertility communication were also reported to have negative implications for people who were not yet partnered, or early on in their relationship trajectories. Fear of rejection when forming new relationships was central to non-partnered participant accounts, particularly among younger cancer survivors who told us, “I am afraid of putting myself out there in case I get rejected” (survivor, woman, 20–25 years, Hodgkin’s Lymphoma) and “In my mind … if I do find someone and it gets to that time and I say, “Oh, I can’t have kids,” they’re just going to get up and go” (Ben, survivor, 15–20 years, testicular). Knowing when to communicate potential infertility when forming new relationships was another significant source of worry for a number of participants, as a survey participant reported, “it worries me. I question whether I should tell the other person and over think it and stress” (survivor, woman, 15–20 years, Ewings Sarcoma). Jasmine (survior, 15–20 years, haematological) said she “wouldn’t know how to bring it up on my own” and Amanda (survivor, 20–25 years, haematological) told us, “its’s not the greatest thing to want to bring up.” Some participants reported that they would “bring it up early” (Nathan, survivor, 20–25 years, Ewing’s Sarcoma) or that they would “tell it to him in the very beginning” (Zoe, survivor, 20–25 years, haematological), as a means to avoid feeling like they are “hiding something, I’m lying” (Tanya, survivor, 35–40 years, gynaecological). Others disclosed that it would “depend on how the relationship is going” (Lucy, survivor, 30–35 years, breast) or that it is “not something you do until you are serious” because “you have got to still remember it could be a big issue … a deal breaker” (Lara, survivor, 20–25 years, haematological). In this vein, some participants reported that they had not discussed fertility within a current relationship as yet, “because we are both quite young” (Roxanne, survivor, 20–25 years, breast), “I’m only 19, so fertility is certainly not something I bring up with potential partners” (survivor, woman, 19 years, Hodgkin’s Lymphoma), or because “we are not thinking about having children together” (Jasmine, survivor, 15–20 years, haematological).

## Discussion

Couple communication is recognised to be central to coping with chronic illness, such as cancer [76], as well as coping with distress associated with infertility [47]. Previous research has focused on general couple communication in the context of cancer [16, 77], or communication about sexual concerns [50, 51]. The present study is the first to examine the nature and perceived quality of couple communication about fertility concerns in the context of cancer, in response to a plea for research in this area [54]. It is also innovative in examining the experiences of both women and men cancer survivors and their partners, across a range of cancer types and age groups, and in the use of in-depth interviews and survey responses.

Through responses to surveys and interviews we identified both positive experiences and challenges surrounding couple communication about cancer related fertility. The majority of participants gave accounts of working together to handle questions about fertility, manifested by openness, honesty, sharing of experiences and listening to each other, which were associated with reports of relationship satisfaction and growth, support, and closeness. This confirms previous findings that mutual constructive communication between intimate partners is associated with relationship satisfaction in the context of both non-cancer related infertility [78], and cancer survivorship [18–20]. It also confirms findings that effective communication can lead to couples finding benefit such as relationship closeness following biographical disruptions resulting from cancer [2, 21] or infertility [47]. Previous research has found that effective couple communication is a significant predictor of sexual functioning after cancer [52], facilitating renegotiation of sex when embodied changes render previous sexual practices difficult [53]. The findings of the present study suggest that effective couple communication may also facilitate coping and adjustment in the context of cancer related fertility concerns.

At the same time as reporting working well together to address fertility concerns, and believing that their partner was comforted by such discussion, a substantial proportion of cancer survivors and partners also agreed that couple communication could be improved. This was reflected in accounts of feeling misunderstood, of self-silencing in order to avoid hurting the other person, and in a smaller proportion of cases, accounts of relationship conflict and fears of separation. These findings suggest that many couples could *both* communicate about cancer related fertility concerns *and* have concerns about the adequacy and impact of such communication. McKenzie-Mohr and Lafrance [79] describe a “both/and” position as challenging the oversimplification of “either/or” binaries, which in the present study, means challenging the conceptualisation of couples as having “satisfactory *or* unsatisfactory” communication, manifested by being either “open *or* with-holding”. Acknowledging that many couples can be *both* open and honest *and* sometimes withhold feelings or feel misunderstood, has implications for oncology practice, as it suggests that a high percentage of couples may benefit from support in improving couple communication about fertility concerns.

There is consistent evidence that oncology health care professionals can facilitate couple communication about difficult psycho-sexual issues associated with cancer, through giving permission for concerns to be addressed, and providing information to facilitate couple coping [80, 81]. The provision of information about the impact of cancer on fertility, as is recommended by clinical guidelines [43, 64], can be the first step in facilitating this discussion. It is recommended that fertility information be provided at the point of diagnosis [43, 64], which will facilitate couples in addressing the “shock” associated with cancer related fertility concerns [38]. However as infertility can be a late effect of cancer, information which serves to facilitate couple communication and coping, as well as decision making in relation to fertility preservation, is also needed after treatment has ended [32, 58, 82].

The provision of fertility information and support has to date focused on cancer survivors on an individual basis [43]. Our findings suggest that the increased attention being paid to couple interventions in general psycho-oncology [1, 13] should be extended to the domain of fertility, with partners included in discussions and interventions as a matter of routine practice. Supportive psycho-social interventions that facilitate couple communication may also be beneficial, serving to “refine” fertility communication ([13] , p. 139). The need for couple-based interventions may be more acute for the minority of participants for whom infertility, or fear of infertility, was reported to be a source of relationship conflict or breakdown, as reported in previous research on infertility in the general population [46]. Infertility can lead to individuals and couples having to re-evaluate life goals, leading to an experience of biographical disruption and psychological distress, which can have an impact on couple relationships [32, 38].

More specifically, couple-based interventions would also be beneficial in addressing practices of self-silencing or holding back through avoiding discussion of infertility concerns. For some, this was to avoid causing their partner distress. Described as protective buffering [83], such self-silencing is undertaken with good intentions, to protect a partner from distress, but it has been found to be associated with decreased relationship satisfaction, increased burden, and poor mental health, in both cancer survivors and their partners [20, 24]. For some, self-silencing was practiced to avoid a subject that was distressing to themselves, a common finding in relation to couple communication about distressing aspects of cancer [84, 85]. Some women rationalised self-silencing in their male partners, drawing on gendered discourses, wherein men are positioned as ‘naturally’ reticent to discuss health concerns and emotionally confronting topics [86]. Whilst there is some evidence that gendered roles may influence patterns of couple communication in the context of cancer [87], the majority of participants in the present study reported that male partners did communicate effectively and provide support. However, gendered patterns of communication do need to be acknowledged in the development of couple-based fertility interventions, to avoid partners not feeling heard or supported. To address this, interventions that adopt a “shared or dyadic approach” are most likely to be appropriate to engage both partners ([13] , p. 142), in both heterosexual and same-sex relationships.

The relational needs and concerns of individuals contemplating fertility discussion in future relationships, or in new relationships where patterns of communication are not yet established, need to be acknowledged and addressed. This is a particular concern for adolescents and young adults (AYAs), for whom cancer related infertility concerns can have a significant impact on quality of life, sexual identity, body image, and confidence in embarking on intimate relationships [60, 70]. Acknowledging that AYAs and older adults may need support in developing strategies for communication about fertility issues with a future partner is an important part of cancer care [29]. Previous research has reported that some oncology health care professionals are reluctant to raise the issue of fertility with AYAs, due to perceived embarrassment on the part of young men, or AYAs expressing disinterest in future fertility [57]. Difficulties in discussing fertility with single people, who are more likely to be AYAs, has also been reported [57]. The provision of written information about the possible impact of cancer on fertility, which includes strategies for communicating about fertility in current and future relationships, is important for both AYAs [88] and older adults [89].

Clinical guidelines recommend discussion of fertility with all cancer patients, to provide information about the impact of cancer treatment and facilitate fertility preservation decision making [43, 64]. This process of information provision can also serve to facilitate couple communication, with positive benefits for couple coping. However, many clinicians are not discussing fertility with their patients [43], resulting in dissatisfaction and distress on the part of cancer survivors and their partners [69]. It is vital that oncology nurses, clinicians and allied health care professionals engage in discussions with their patients about the of the impact of cancer on fertility, and include partners where this is appropriate. As many health care professionals do not feel equipped to engage in this discussion, due to lack of training and experience [57], there is a clear need for professional development about the impact of cancer on fertility, the fertility preservation options open to patients and their partners, and the most effective means of communicating fertility information [29, 43, 57]. Examination of how healthcare professionals incorporate partners into their conversations about fertility, and the impact of including partners, is an area in need of further investigation. Psychological support for couples in communicating about fertility concerns, and for individuals who are concerned about future relationships, may have a positive impact on psychological wellbeing and relationship functioning, as has been found in other areas of oncology [13, 20]. Further research is needed to assess the efficacy of different models of couple intervention and their impact on adaptation to compromised fertility after cancer.

This study had a number of strengths and limitations. The strengths were the use of a survey with a large sample of men and women cancer survivors and partners, across cancer types and age groups, and qualitative interviews to examine subjective accounts of couple communication about fertility issues in depth. Further research is needed to systematically explore how patterns of couple communication interact with relationship satisfaction, wellbeing and coping with fertility concerns, across tumour types, in order to have a clear picture of which couples may be most at risk of experiencing difficulties and develop strategies to address concerns. The cross sectional nature of the data is a limitation; future research using a longitudinal design could usefully examine couple communication at different stages of cancer experience. The smaller number of men, and primarily heterosexual nature of the sample is also a limitation. A greater number of men, and a more gender and sexuality diverse sample would facilitate understanding of the experience of LGBTQ cancer survivors and their partners, recognised to be an invisible diversity within cancer research and services [90], often and an neglected group in the provision of information about fertility from health care professionals [57].

## Conclusions

In this paper we have identified the nature and perceived quality of couple communication in addressing fertility concerns associated with cancer. Our findings reinforce the notion of cancer as a ‘we-disease’ [2] and the dyadic nature of cancer coping, identifying the importance of a relational perspective when addressing and providing support for fertility concerns. There is a need for partners to be incorporated into clinical discussions of cancer related infertility, as well as fertility preservation, in order to provide information to couples, and facilitate couple communication about fertility concerns. Couple interventions developed in general psycho-oncology should be extended to the domain of fertility, in order to facilitate effective couple communication. Identification of difficulties in couple communication, and provision of support to enhance such communication, has the potential to have positive impacts on both couple relationships and coping with cancer related infertility concerns. At the same time, communication in future relationships needs to be addressed for single people and adolescents and young adults (AYAs) who may have fertility concerns.

## Supplementary Information

**Additional file 1.** Interview schedule

**Additional file 2.** Survey

**Additional file 3: Table S3**. Impact of fertility concerns on relationships by gender for cancer survivors, FPI items.

**Additional file 4: Table S4.** Impact of fertility concerns on relationships by gender for partners of people with cancer, FPI items.

## Data Availability

The datasets analysed during the current study are available from the corresponding author on reasonable request.

## Abbreviations

AYA: Adolescents and young adults with cancer
FPI: Fertility Preservation Inventory
LGBTQ: Lesbian, gay, bisexual, transgender, queer
SD: Standard deviation
