# Supplementary material for: Talking but not always understanding: couple communication about infertility concerns after cancer
Source: BMC Public Health. 2021 Jan 19;21:161. doi: 10.1186/s12889-021-10188-y (PMC7816453; doi:10.1186/s12889-021-10188-y)
Supplement: Supplementary file 1 — Additional file 1. Interview schedule [file 12889_2021_10188_MOESM1_ESM.pdf]

<u>PWC/Partner Interview Schedule</u>

- **Preamble**

Thanks very much for agreeing to do this interview as part of the Cancer and Fertility Study. One of the outcomes of this study is that we hope to find better ways to support people who are living with cancer and its effects on their fertility.

You may remember from the information that we gave you what we'll be covering during the interview. The topics we'll talk about will be about your experience of having cancer and the effect it has had on your fertility, your wellbeing, your relationship with your partner (or feelings you have about a new relationship) and your day-to-day life. We'll also talk about any support and information that you have had or that you would find or have found useful.

The interview will take about an hour and as mentioned on the consent form it will be recorded so we can go over what you said later. I just want to emphasise that anything you say is completely confidential. You don't have to answer any questions that you feel uncomfortable answering and you can stop the interview at any time to take a break or stop altogether.

Do you have any questions before we start?

CONFIRM STATUS
e.g You said in your survey that you are….(married, with one child who was born before your cancer diagnosis… you were diagnosed with early stage breast cancer in 2009 …)

- **Construction / meaning of fertility**

- **Experience of fertility in the context of cancer**

1. You mentioned in the survey that there have been some issues around fertility since the diagnosis of cancer. Can you talk a little bit more about these issues?
   - Prompt:
     - Before and after cancer, what happened?
     - Treatment decision
     - Meaning, understanding

2. How have these made you feel?
   - Prompt:

- Personal – self, identity, psychologically, sexuality, body image, physically, premature menopause if appropriate
- Relational –relationship (intimacy, sexual), future relationships if not partnered

3. How has this affected your partner?

- **Negotiation**

4. Can you tell me about your experience of talking with your partner (potential partners) about fertility?
    - Prompt
    - How did/do you feel talking about it
    - What made it easy or hard
    - Talking to future partners (young people, single people)

5. Can you tell me about what you have tried to deal with the changes to fertility?
    - Prompt:
        - Other options (e.g., IVF)
        - Have you made any changes to your life as a result (e.g., adoption, career change)

6. Can you tell me if the situation has changed over time? How has it changed?

7. Have your feelings about your fertility issues changed over time?

8. (if appropriate ask) How do you feel about becoming pregnant or having a baby in the future?

9. How do you feel when other people you know are pregnant or have a baby?

- **Support**

10. Can you talk about the types of support and information you've received from health professionals in relation to fertility issues?
    - Prompt:
        - Positive and negative experiences
        - Difficulties
        - How and when was it raised? Who raised it?
        - How did this support and information affect the way you coped with your fertility issues?
        - Do you feel anything about you affected the way health professionals provided information and support (e.g., age, relationship status, sexual orientation, occupation)

11. What about support from partner/ family/friends/peers with cancer? Can you talk to me about that?

12. What are the kinds of support and information you would have liked (think are needed)?
    - Prompts:
        - Whose responsibility is it to provide these supports?
        - When do you think support around fertility should be raised?

- **Closing questions**

13. From your experience what would you say to a woman/man who has just been diagnosed with cancer regarding her/his fertility?

14. Is there anything else about your experience that you would like to talk about or you think we haven't covered?

- **Closing statement**

Thank you for taking the time to share your experience with me.
(If appropriate ask) We would be very interested to hear your partner's experience of the fertility issues you have discussed if they were happy to complete the survey too.
