# Supplementary material for: Talking but not always understanding: couple communication about infertility concerns after cancer
Source: BMC Public Health. 2021 Jan 19;21:161. doi: 10.1186/s12889-021-10188-y (PMC7816453; doi:10.1186/s12889-021-10188-y)
Supplement: Supplementary file 2 — Additional file 2. Survey [file 12889_2021_10188_MOESM2_ESM.pdf]

ID: ____________________

# CANCER & FERTILITY STUDY

**As part of the Cancer & Fertility Study being conducted at the University of Western Sydney, we are interested in your experiences of fertility in the context of cancer and your overall health and wellbeing.**

**Taking part in this study is voluntary. You don't have to complete this survey if you don't want to.**

**If you are in a relationship, we are also interested in the experiences of your partner. Your partner can complete a separate survey.**

## Instructions

- The survey will take about 30-45 minutes to complete.
- Please answer all questions.
- Please read the instructions for each set of questions before answering.
- There are no right or wrong answers, just choose the answer that is right for you.
- The information you provide is confidential so no one will know you have taken part in the study.
- Space is provided at the end of the survey for any other comments you may want to add.

**Your participation in this study is greatly appreciated.**

## Section 1: General questions about you

1. **What is today's date?** (dd/mm/yyyy)
___ ___ / ___ ___ / **2 0** ___ ___

2. **What is your age?** __________ yrs

3. **Where did you find out about this study?** *(please tick as many as appropriate)*
   - ☐ Cancer Council NSW
   - ☐ National Breast Cancer Foundation/Register4
   - ☐ CanTeen
   - ☐ Family Planning NSW
   - ☐ Westmead Hospital
   - ☐ Nepean Hospital
   - ☐ University of Western Sydney
   - ☐ BCNA
   - ☐ Newsletter
   - ☐ Cancer Support Groups
   - ☐ Other (please specify) _____________

4. **Are you**
   - ☐ A **PERSON** who has/had cancer
   - ☐ A **PARTNER** of a person who has/had cancer
   - ☐ Both my partner **AND** I have/had cancer

5. **What is your gender?**
   - ☐ Male ☐ Female
   - ☐ I identify as _______________________

6. **With which ethnic or cultural group do you identify?**
______________________________________

7. **With which religious group do you identify?**
______________________________________

8. **What is your Australian postcode?** ________

   If you live overseas, please specify country:
   ______________________________________

9. **How would you describe where you live?**
   - ☐ Metropolitan (e.g., major city) ☐ Regional
   - ☐ Rural ☐ Remote

10. **What is the highest level of education you have achieved?**
    - ☐ Still in school
    - ☐ Left school with no school certificate
    - ☐ School Certificate / HSC
    - ☐ Trade / apprenticeship qualification / certificate / diploma
    - ☐ University degree or higher
    - ☐ Other (Please specify)
    ______________________________
    __

11. **What is your employment status?**
    - ☐ Student ☐ Home duties
    - ☐ Working Full-time ☐ Working Part-time
    - ☐ Casual Work ☐ Volunteer
    - ☐ On leave from work ☐ Unemployed (looking for work)
    - ☐ Not working / Retired
    - ☐ Other (please specify)
    __________________________________

    **If working,**

    **a)** On average, how many hours of paid work do you do per week? __________ hrs

    **b)** On average, how many hours of unpaid/volunteer work outside the home do you do per week? __________ hrs

12. **Do you identify as:**
    - ☐ Heterosexual (i.e., straight) ☐ Non-Heterosexual (e.g., gay, lesbian)
    - ☐ I identify as _______________________

**13. What is your current relationship status?**

- ☐ Have **never** been in a relationship
- ☐ Not in a relationship – The relationship ended **before** the cancer diagnosis
- ☐ Not in a relationship – The relationship ended **after** the cancer diagnosis
- ☐ Not in a relationship – Partner has passed away
- ☐ In a relationship - The relationship started **before** the cancer diagnosis
- ☐ In a relationship - The relationship started **after** the cancer diagnosis
- ☐ Other (please specify) ________________________________

**a)** How long have you been in this relationship OR how long ago was your last relationship?__________ (yrs/mths)

**14. Do you have children?**

☐ Yes ☐ No

**If NO, please go to Q.15.**

**a)** If YES, are they *(please tick both if appropriate)*

- ☐ Biological children
- ☐ Other (please specify): ______________________________ __

**b)** If they are your biological children, were any conceived after the cancer diagnosis?

☐ Yes ☐ No ☐ N/A

**c)** How many children do you have? _______

**d)** What are their gender and ages?

| **Gender (M/F)** | | | | | | |
|---|---|---|---|---|---|---|
| **Age (yrs)** | | | | | | |

**15. Prior to the cancer diagnosis, were you planning to have children or more children?**

☐ Yes ☐ No

## Section 2: Questions about your general wellbeing

**16. How would you rate your quality of life?** (please put a 'X' on the line below)

Worst imaginable |—|—|—|—|—|—|—|—|—|—| Perfect

**The following questions (Q.17-Q.26) concern how you have been feeling recently, please tick the box that best represents how you have been.**

| | All of the time | Most of the time | Some of the time | A little of the time | None of the time |
|---|---|---|---|---|---|
| **17. How often do you feel tired for no good reason?** | | | | | |
| **18. How often do you feel nervous?** | | | | | |
| **19. How often do you feel so nervous that nothing could calm you down?** | | | | | |
| **20. How often do you feel hopeless?** | | | | | |
| **21. How often do you feel restless or fidgety?** | | | | | |
| **22. How often do you feel so restless you could not sit still?** | | | | | |

| | All of the time | Most of the time | Some of the time | A little of the time | None of the time |
|---|---|---|---|---|---|
| **23. How often do you feel depressed?** | | | | | |
| **24. How often do you feel that everything is an effort?** | | | | | |
| **25. How often do you feel so sad that nothing can cheer you up?** | | | | | |
| **26. How often do you feel worthless?** | | | | | |

## Section 3: Questions about your cancer experience

**Note: If both you and your partner have/had cancer, please complete the following questions from your perspective.**

**27. In which year were you or your partner diagnosed with cancer?**

_______________________________________

**28. What type of cancer were you or your partner diagnosed with?**

_______________________________________

**29. At what stage was the cancer when it was first diagnosed?**

- ☐ Early
- ☐ Advanced
- ☐ Unsure
- ☐ Other

If Other, please specify

_______________________________________

_______________________________________

**30. Have you or your partner been diagnosed with another PRIMARY cancer (not your cancer coming back) since the first diagnosis?**

- ☐ Yes
- ☐ No

**If YES,**

**a)** What was the cancer?

_______________________________________

**b)** In which year was it diagnosed?

_______________________________________

**31. What has happened to the cancer(s) since it was diagnosed?**

| 1st Cancer | 2nd Cancer | |
|---|---|---|
| ☐ | ☐ | Currently being treated |
| ☐ | ☐ | The cancer was cured (no evidence of cancer at the moment) |
| ☐ | ☐ | The cancer came back and was cured again |
| ☐ | ☐ | The cancer came back and is being treated again |
| ☐ | ☐ | The cancer came back and is being monitored at the moment |
| ☐ | ☐ | No further treatment possible / palliative care |
| ☐ | ☐ | Other (please specify) ____________________ ____________________ |

**32. What treatments have you or your partner received for the cancer(s) in the past and now?** *(please tick as many as appropriate)*

| Past | Now | |
|---|---|---|
| ☐ | ☐ | No Treatments |
| ☐ | ☐ | Surgery *(e.g., mastectomy, prostatectomy, hysterectomy)* |
| ☐ | ☐ | Radiotherapy *(e.g., external beam, brachytherapy)* |
| ☐ | ☐ | Chemotherapy |
| ☐ | ☐ | Hormonal treatment *(e.g., Tamoxifen or similar tablets, Zoladex implants, Lucrin injections)* |
| ☐ | ☐ | Complementary Medicine *(e.g., naturopathy, acupuncture, herbal remedies, yoga)* |
| ☐ | ☐ | I am not sure |
| ☐ | ☐ | Other (please specify) ______________________ ______________________ |

**33. Have you, or your woman partner, experienced menopause as a result of cancer or cancer treatment?**

☐ Yes ☐ No ☐ N/A

**If YES**, please clarify

__________________________________________

__________________________________________

__________________________________________

__________________________________________

__________________________________________

__________________________________________

__________________________________________

__________________________________________

__________________________________________

__________________________________________

**34. Some cancer diagnoses might be associated with a genetic mutation, have you or your partner been offered genetic testing?**

☐ Yes ☐ No

a) **If YES**, did you or your partner have the test?

☐ Yes ☐ No

b) **If YES**, have you or your partner been found to have a genetic mutation?

☐ Yes ☐ No result yet

☐ Inconclusive / negative

**The following statements (Q.35-Q.42) are about how your or your partner's cancer has affected <u>you</u>.** *Please circle the best response for you. There are <u>no right answers</u> to any of the questions.*

| | Strongly Agree | | | | Strongly Disagree | N/A |
|---|---|---|---|---|---|---|
| **35. I have a hard time adjusting to the limitations of cancer.** | **1** | **2** | **3** | **4** | **5** | **X** |
| **36. Because of cancer, I miss the things I like to do most.** | **1** | **2** | **3** | **4** | **5** | **X** |
| **37. Cancer makes me feel useless at times.** | **1** | **2** | **3** | **4** | **5** | **X** |
| **38. Cancer makes me more dependent on others than I want to be.** | **1** | **2** | **3** | **4** | **5** | **X** |
| **39. Cancer makes me a burden on my family and friends.** | **1** | **2** | **3** | **4** | **5** | **X** |
| **40. Cancer does not make me feel inadequate.** | **1** | **2** | **3** | **4** | **5** | **X** |
| **41. I will never be self-sufficient enough to make me happy.** | **1** | **2** | **3** | **4** | **5** | **X** |
| **42. I think people are often uncomfortable being around me because of cancer.** | **1** | **2** | **3** | **4** | **5** | **X** |

## Section 4: Questions about fertility

**43. Were you concerned about fertility <u>BEFORE</u> the cancer diagnosis?** *(please put a 'X' on the line below)*

|_____|_____|_____|_____|_____|_____|_____|_____|_____|

**Not at all concerned** ... **Very concerned**

Please comment

______________________________________________________________________

______________________________________________________________________

______________________________________________________________________

**44. Were you concerned about fertility <u>AFTER</u> the cancer diagnosis?** *(please put a 'X' on the line below)*

|_____|_____|_____|_____|_____|_____|_____|_____|_____|

**Not at all concerned** ... **Very concerned**

Please comment

______________________________________________________________________

______________________________________________________________________

______________________________________________________________________

**45. Did your concerns about fertility affect the decision(s) you made about cancer treatments?**

☐ Yes ☐ No

Please comment

______________________________________________________________________

______________________________________________________________________

______________________________________________________________________

**The following (Q.46-Q.73) are experiences that some people have with fertility and parenthood, please tick the box which best answers that item for you.** <u>*Note*</u>*: Please use 'Non-Applicable (N/A)' for experiences that are not relevant to you.*

| | Strongly Disagree | Disagree | Mildly Disagree | Mildly Agree | Agree | Strongly Agree | N/A |
|---|---|---|---|---|---|---|---|
| **46. Pregnancy and childbirth are the two most important events for a person.** | | | | | | | |
| **47. It doesn't bother me when I'm asked questions about children.** | | | | | | | |
| **48. People without a child are just as happy as those with children.** | | | | | | | |

| | | Strongly Disagree | Disagree | Mildly Disagree | Mildly Agree | Agree | Strongly Agree | N/A |
|---|---|---|---|---|---|---|---|---|
| 49. | For me, being a parent is a more important goal than having a satisfying career. | | | | | | | |
| 50. | Family members don't seem to treat me any differently. | | | | | | | |
| 51. | I could see a number of advantages if I didn't have a child (or another child). | | | | | | | |
| 52. | Relationships need a child. | | | | | | | |
| 53. | The holidays are especially difficult for me. | | | | | | | |
| 54. | I could visualise a happy life without a child (or another child). | | | | | | | |
| 55. | It's hard to feel like a true adult until you have a child. | | | | | | | |
| 56. | Family get-togethers are especially difficult for me. | | | | | | | |
| 57. | At times, I seriously wonder if I want a child (or another child). | | | | | | | |
| 58. | A future without a child (or another child) would frighten me. | | | | | | | |
| 59. | I can't help comparing myself with friends who have children. | | | | | | | |
| 60. | Not having a child (or another child) would allow me time to do other satisfying things. | | | | | | | |
| 61. | I feel empty because of fertility issues. | | | | | | | |
| 62. | I still have lots in common with friends who have children. | | | | | | | |
| 63. | Having a child (or another child) is not necessary for my happiness. | | | | | | | |
| 64. | Having a child (or another child) is not the major focus of my life. | | | | | | | |
| 65. | I find it hard to spend time with friends who have young children. | | | | | | | |

| | | | | | | | |
|---|---|---|---|---|---|---|---|
| **66.** **I could have a long, happy relationship without a child (or another child).** | | | | | | | |
| **67.** **I have often felt that I was born to be a parent.** | | | | | | | |
| | **Strongly Disagree** | **Disagree** | **Mildly Disagree** | **Mildly Agree** | **Agree** | **Strongly Agree** | **N/A** |
| **68.** **When I see families with children I feel left out.** | | | | | | | |
| **69.** **There is a certain freedom without children that appeals to me.** | | | | | | | |
| **70.** **As long as I can remember, I've wanted to be a parent.** | | | | | | | |
| **71.** **I feel like friends or family are leaving us behind.** | | | | | | | |
| **72.** **I will do just about anything to have a child (or another child).** | | | | | | | |
| **73.** **It doesn't bother me when others talk about their children.** | | | | | | | |

**74.** **Have fertility issues affected your relationship(s) or your ability to form a new relationship?**

☐ Yes ☐ No

Please comment

______________________________________________________________________

______________________________________________________________________

______________________________________________________________________

______________________________________________________________________

______________________________________________________________________

**75.** **Have fertility issues affected your feelings about your body?**

☐ Yes ☐ No

Please comment

______________________________________________________________________

______________________________________________________________________

______________________________________________________________________

______________________________________________________________________

______________________________________________________________________

**76.** **Have fertility issues affected your feelings about yourself as a man or a woman?**

☐ Yes ☐ No

Please comment

______________________________________________________________________

______________________________________________________________________________
______________________________________________________________________________
______________________________________________________________________________
______________________________________________________________________________

## Section 5: Questions relating to information & support about cancer & fertility

**77. Since receiving the cancer diagnosis, have issues about fertility been discussed with a health care professional?**

☐ Yes ☐ No

**If YES,**

**a)** Which health care professional(s)?

______________________________

**b)** Who raised the issues?

- ☐ Health care professional
- ☐ Yourself
- ☐ Your partner
- ☐ Both you and your partner
- ☐ Other (please specify)

______________________________
______________________________
____

**c)** How satisfied were you with the discussion?

☐ Satisfied ☐ Dissatisfied
☐ Neither satisfied nor dissatisfied

Please tell us more about your response

______________________________
______________________________
______________________________
______________________________
______________________________

**78. If issues about fertility <u>have not</u> been discussed, would you have liked a health care professional to raise the issues?** *(Please go to Q.79 if you had responded 'Yes' to Q.77)*



☐ Yes ☐ No

If YES, which health care professional(s) would you like to raise the issue?

______________________________
______________________________

**79. When do you think issues relating to cancer and fertility should be discussed by a health care professional?**

- ☐ At the time of diagnosis
- ☐ At the commencement of treatment
- ☐ Post-treatment and during recovery
- ☐ At each stage
- ☐ Never
- ☐ Other (Please specify)

______________________________
__

**80. Have you used or received information/resources on cancer and fertility?**

☐ Yes ☐ No

**If YES,**

**a)** What were the information/resources?

______________________________
______________________________
______________________________
______________________________

**b)** How were the information/resources helpful or unhelpful?

______________________________
______________________________

**81. Would you like to receive information about the effects of cancer and treatments on fertility?**

☐ Yes ☐ No

**a) If YES**, and this were available, how would you like to receive this information? *(please tick as many as appropriate)*

☐ Booklet/Leaflet
☐ Newsletter
☐ Telephone Helpline
☐ Websites
☐ Group/Information Sessions
☐ Face-to-face, individual consultation
☐ Other (please specify)

**82. Have you had any fertility investigations or fertility preserving procedures** *(e.g., sperm banking, embryo cryopreservation, trachelectomy)***?**

☐ Yes ☐ No

**If YES**, please describe your experience of these investigations and/or undergoing these procedures both BEFORE (if appropriate) and AFTER (if appropriate) the cancer diagnosis.



**83. Have you attempted to become pregnant since the cancer diagnosis?**

☐ Yes ☐ No

**a) If YES**, have you been successful in becoming pregnant?

☐ Yes ☐ No

Please comment

**b) If NO**, do you currently have any plans to attempt to become pregnant?

☐ Yes ☐ No

Please comment

**84. Have you spoken with someone about fertility issues?**

☐ Yes ☐ No

**If NO**, please comment

_______________________________________

_______________________________________

_______________________________________

_______________________________________

_______________________________________

_______________________________________

**If YES, please continue to Q.85.**

**If NO, please go to Section 6 – Q.90.**

**85. If you have spoken with someone about fertility issues, who have you spoken to? (Tick all that apply) Also, please indicate how satisfied you were with the discussion.**

| | Have spoken to | Satisfied | Neither satisfied or dissatisfied | Not satisfied |
|---|---|---|---|---|
| Partner (husband, wife, boyfriend, girlfriend) | ☐ | ☐ | ☐ | ☐ |
| Friend / Relative | ☐ | ☐ | ☐ | ☐ |
| GP | ☐ | ☐ | ☐ | ☐ |
| Medical oncologist | ☐ | ☐ | ☐ | ☐ |
| Radiation oncologist | ☐ | ☐ | ☐ | ☐ |
| Surgeon | ☐ | ☐ | ☐ | ☐ |
| Fertility Specialist | ☐ | ☐ | ☐ | ☐ |
| Nurse | ☐ | ☐ | ☐ | ☐ |
| Social Worker | ☐ | ☐ | ☐ | ☐ |
| Counsellor | ☐ | ☐ | ☐ | ☐ |
| Psychologist | ☐ | ☐ | ☐ | ☐ |
| Religious / pastoral care worker | ☐ | ☐ | ☐ | ☐ |
| Relationship therapist | ☐ | ☐ | ☐ | ☐ |
| Person affected by cancer | ☐ | ☐ | ☐ | ☐ |
| Support Group | ☐ | ☐ | ☐ | ☐ |
| Cancer support helpline | ☐ | ☐ | ☐ | ☐ |
| Other (please specify) 1.______________________________ | ☐ | ☐ | ☐ | ☐ |
| Other (please specify) 2.______________________________ | ☐ | ☐ | ☐ | ☐ |

**86. Please explain what was satisfying, or dissatisfying, about discussing fertility issues with your partner, family and friends.**

_______________________________________________________________________________

_______________________________________________________________________________

_______________________________________________________________________________

_______________________________________________________________________________

_______________________________________________________________________________

**87. Please explain what was satisfying, or dissatisfying, about discussing fertility issues with <u>people who have, or have had, cancer</u>.**

________________________________________________________________________

________________________________________________________________________

________________________________________________________________________

________________________________________________________________________

________________________________________________________________________

**88. Please explain what was satisfying, or dissatisfying, about discussing fertility issues with <u>health care professionals</u> (e.g., specialists, doctors, nurses, social workers, psychologists) who are caring or have cared for you or your partner.**

________________________________________________________________________

________________________________________________________________________

________________________________________________________________________

________________________________________________________________________

________________________________________________________________________

**89. Do you have any other comments you would like to make about your experience in talking about fertility issues with others?**

________________________________________________________________________

________________________________________________________________________

________________________________________________________________________

________________________________________________________________________

________________________________________________________________________

## Section 6: Questions about your relationship with your intimate partner

**The following sections are about how fertility issues caused by cancer can affect a person's intimate and sexual relationship with his/her partner. If you are <u>under 18 years old</u>, <u>not sexually active</u> or <u>not in an intimate couple relationship</u>, some of these sections might not be relevant for you.**

**The following questions (Q.90-Q.111) concern your feelings about the relationship with your partner. Please circle the number which best answers that item for you.** *Please use 'Non-Applicable (N/A)' for experiences that are not relevant to you.*

**<u>Note</u>: If all these questions are irrelevant to you, please go to Q.112.**

| | | | | | | | |
|---|---|---|---|---|---|---|---|
| 90. | How well does your partner meet your needs? | 1<br>Poorly | 2 | 3<br>Average | 4 | 5<br>Extremely well | 0<br>N/A |
| 91. | In general, how satisfied are you with your relationship? | 1<br>Unsatisfied | 2 | 3<br>Average | 4 | 5<br>Extremely satisfied | 0<br>N/A |
| 92. | How good is your relationship compared to most? | 1<br>Poor | 2 | 3<br>Average | 4 | 5<br>Excellent | 0<br>N/A |
| 93. | How often do you wish you weren't in this relationship? | 5<br>Never | 4 | 3<br>Average | 2 | 1<br>Very often | 0<br>N/A |
| 94. | To what extent has your relationship met your original expectations? | 1<br>Hardly at all | 2 | 3<br>Average | 4 | 5<br>Completely | 0<br>N/A |
| 95. | How much do you love your partner? | 1<br>Not much | 2 | 3<br>Average | 4 | 5<br>Very much | 0<br>N/A |
| 96. | How many problems are there in your relationship? | 5<br>Very Few | 4 | 3<br>Average | 2 | 1<br>Very many | 0<br>N/A |

**Most people have disagreements in their relationships.** *Please tick the box which best answers that item for you.*

| | | All the time | Most of the time | More often than not | Occasionally | Rarely | Never | N/A |
|---|---|---|---|---|---|---|---|---|
| 97. | How often do you discuss or have you considered divorce, separation, or terminating your relationship? | | | | | | | |
| 98. | How often do you or your partner leave the house after a fight? | | | | | | | |
| 99. | In general, how often do you think that things between you and your partner are going well? | | | | | | | |
| 100. | Do you confide in your partner? | | | | | | | |
| 101. | Do you ever regret that you married/have a partner (or lived together)? | | | | | | | |
| 102. | How often do you and your partner argue? | | | | | | | |
| 103. | How often do you and your partner "get on each other's nerves?" | | | | | | | |

**How would you rate the following?** *Please tick the box which best answers that item for you.*

| | Every day | Almost every day | Occasionally | Rarely | Never | N/A |
|---|---|---|---|---|---|---|

| | | | | | | |
|---|---|---|---|---|---|---|
| **104. Do you kiss your partner?** | | | | | | |
| | **All of them** | **Most of them** | **Some of them** | **Very few of them** | **None of them** | **N/A** |
| **105. Do you and your partner have outside interests together?** | | | | | | |

**How often do the following occur between you and your partner?** *Please tick the box which best answers that item for you.*

| | Never | Less than once a month | Once or twice a month | Once or twice a week | Once a day | More often | N/A |
|---|---|---|---|---|---|---|---|
| **106. Have a stimulating exchange of ideas** | | | | | | | |
| **107. Laugh together** | | | | | | | |
| **108. Calmly discuss something** | | | | | | | |
| **109. Work together on a project** | | | | | | | |

**110. The following table represents different degrees of happiness in a relationship.** *Please tick the box that you think represents your relationship.*

| Extremely unhappy | Fairly unhappy | A little unhappy | Happy | Very happy | Extremely happy | Perfect |
|---|---|---|---|---|---|---|
| | | | | | | |

☐ **Not in a relationship**

**111. Please circle the number next to the statement that best describes how you feel about the future of your relationship.**

| | |
|---|---|
| **5** | I want desperately for my relationship to succeed and **would go to almost any length** to see that it does. |
| **4** | I want very much for my relationship to succeed and **will do all I can** to see that it does. |
| **3** | I want very much for my relationship to succeed and **will do my fair share** to see that it does. |
| **2** | It would be nice if my relationship succeeded, and **I can't do much more than I am doing now** to help it succeed. |
| **1** | It would be nice if it succeeded, but **I refuse to do any more than I am doing now** to keep the relationship going. |
| **0** | My relationship can never succeed, and **there is no more that I can do** to keep the relationship going. |
| **N/A** | Not in a relationship. |

**The following statements (Q.112-Q.129) are about how concerns over fertility issues can affect a person's sexual and intimate relationship with his/her partner, please tick the box which best answers that item for you.** *Please use 'Non-Applicable (N/A)' for experiences that are not relevant to you.*

**Note: If all these statements are irrelevant to you, please go to Q.130.**

| | Strongly Disagree | Disagree | Mildly Disagree | Mildly Agree | Agree | Strongly Agree | N/A |
|---|---|---|---|---|---|---|---|
| **112. I can't show my partner how I feel because it will make him/her feel upset.** | | | | | | | |
| **113. When I talk about our fertility issues, my partner seems comforted by my comments.** | | | | | | | |
| **114. My partner doesn't understand the way fertility issues affect me.** | | | | | | | |
| **115. I find I've lost my enjoyment of sex because of fertility issues.** | | | | | | | |
| **116. My partner and I work well together handling questions about our infertility.** | | | | | | | |
| **117. I feel just as attractive to my partner as before.** | | | | | | | |
| **118. It bothers me that my partner reacts differently to our fertility issues.** | | | | | | | |
| **119. I don't feel any different from other members of my sex.** | | | | | | | |
| **120. My partner is quite disappointed with me.** | | | | | | | |
| **121. I feel like I've failed at sex.** | | | | | | | |
| **122. My partner and I *could* talk more openly with each other about our fertility issues.** | | | | | | | |
| **123. During sex, all I can think about is wanting a child (or another child).** | | | | | | | |
| **124. I couldn't imagine us ever separating because of fertility issues.** | | | | | | | |
| **125. Having sex is difficult because I don't want another disappointment.** | | | | | | | |
| **126. When I try to talk about fertility issues, it seems to lead to an argument.** | | | | | | | |
| **127. If I miss a critical day to have sex, I can feel quite angry.** | | | | | | | |

| | Strongly Disagree | Disagree | Mildly Disagree | Mildly Agree | Agree | Strongly Agree | N/A |
|---|---|---|---|---|---|---|---|
| **128. Because of infertility, I worry that my partner and I are drifting apart.** | | | | | | | |
| **129. Sometimes I feel so much pressure, that having sex becomes difficult.** | | | | | | | |

**130. Do you have any other issues around cancer and fertility that you would like to comment upon?**

______________________________________________________________________________

______________________________________________________________________________

______________________________________________________________________________

______________________________________________________________________________

______________________________________________________________________________

______________________________________________________________________________

______________________________________________________________________________

______________________________________________________________________________

______________________________________________________________________________

______________________________________________________________________________

______________________________________________________________________________

______________________________________________________________________________

______________________________________________________________________________

______________________________________________________________________________

______________________________________________________________________________

______________________________________________________________________________

______________________________________________________________________________

______________________________________________________________________________

______________________________________________________________________________

______________________________________________________________________________

______________________________________________________________________________

______________________________________________________________________________

______________________________________________________________________________

______________________________________________________________________________

______________________________________________________________________________

______________________________________________________________________________

**This is the end of the survey.**
**Thank you very much for your time and contribution to the study.**

If you have some discomfort or experience any strong emotions after completing the survey, and feel as though you would like to talk to someone, please feel free to contact a member of the research team (☏ 1800 19 20 02). Alternatively, please see below for some numbers that you can call for help, trained staff are ready to take your call and provide the appropriate service to you.

- Cancer Council Helpline (☏ 13 11 20) – Monday to Friday, 9am-5pm
- Lifeline Australia (☏ 13 11 14) – 24 hour service
- The Kids Helpline (☏ 1800 55 1800) – this is a telephone service for young people aged 5-25 years

If you are a Member of CanTeen, you can talk to your Member Liaison Officer (MLO).
