# Supplementary material for: Talking but not always understanding: couple communication about infertility concerns after cancer
Source: BMC Public Health. 2021 Jan 19;21:161. doi: 10.1186/s12889-021-10188-y (PMC7816453; doi:10.1186/s12889-021-10188-y)
Supplement: Supplementary file 3 — Additional file 3: Table S3. Impact of fertility concerns on relationships by gender for cancer survivors, FPI items. [file 12889_2021_10188_MOESM3_ESM.docx]

Supplementary Table 3. Impact of fertility concerns on relationships by gender for cancer survivors, FPI items

| **Statement** | **People with cancer** | | | |  | | |
| --- | --- | --- | --- | --- | --- | --- | --- |
|  | **Women** | | **Men** | | **Test for group difference** | | |
|  | ***n*** | **%** | ***n*** | **%** | ***χ^2^*** | ***p*** | $\boldsymbol{\emptyset}$ |
| I can’t show my partner how I feel because it will make him/her feel upset |  |  |  |  |  |  |  |
| Agree  Disagree | 118  258 | 31.4  68.6 | 31  58 | 34.8  65.2 | .393 | .531 | .029 |
| When I talk about our fertility issues, my partner seems comforted by my comments |  |  |  |  |  |  |  |
| Agree  Disagree | 293  80 | 77.7  21.1 | 73  16 | 78.5  17.2 | 5.196 | .074 | .105 |
| My partner doesn’t understand the way fertility issues affect me |  |  |  |  |  |  |  |
| Agree  Disagree | 100  274 | 26.7  73.3 | 18  71 | 20.2  79.8 | 1.606 | .205 | .059 |
| My partner and I work well together handling questions about our infertility |  |  |  |  |  |  |  |
| Agree  Disagree | 343  31 | 91.7  8.3 | 72  17 | 80.9  19.1 | 9.045 | .003 | .140 |
| It bothers me that my partner reacts differently to our fertility issues |  |  |  |  |  |  |  |
| Agree  Disagree | 75  299 | 20.1  79.9 | 10  79 | 11.2  88.8 | 3.729 | .053 | .090 |
| My partner and I could talk more openly with each other about our fertility issues |  |  |  |  |  |  |  |
| Agree  Disagree | 240  132 | 64.5  35.5 | 64  25 | 71.9  28.1 | 1.748 | .186 | .062 |
| I couldn’t imagine us ever separating because of fertility issues |  |  |  |  |  |  |  |
| Agree  Disagree | 322  51 | 86.3  13.7 | 75  14 | 84.3  15.7 | .252 | .616 | .023 |
| When I try to talk about fertility issues, it seems to lead to an argument |  |  |  |  |  |  |  |
| Agree  Disagree | 26  348 | 7.0  93.0 | 8  81 | 9.0  91.0 | .438 | .508 | .031 |
| Because of infertility, I worry that my partner and I are drifting apart |  |  |  |  |  |  |  |
| Agree  Disagree | 31  342 | 8.3  91.7 | 8  81 | 9.0  91.0 | .043 | .836 | .010 |
