# Supplementary material for: Talking but not always understanding: couple communication about infertility concerns after cancer
Source: BMC Public Health. 2021 Jan 19;21:161. doi: 10.1186/s12889-021-10188-y (PMC7816453; doi:10.1186/s12889-021-10188-y)
Supplement: Supplementary file 4 — Additional file 4: Table S4. Impact of fertility concerns on relationships by gender for partners of people with cancer, FPI items. [file 12889_2021_10188_MOESM4_ESM.docx]

Supplementary Table 4. Impact of fertility concerns on relationships by gender for partners of people with cancer, FPI items

| **Statement** | **Partners of people with cancer** | | | |  | | |
| --- | --- | --- | --- | --- | --- | --- | --- |
|  | **Women** | | **Men** | | **Test for group difference** | | |
|  | ***n*** | **%** | ***n*** | **%** | ***χ^2^*** | ***p*** | $\boldsymbol{\emptyset}$ |
| I can’t show my partner how I feel because it will make him/her feel upset |  |  |  |  |  |  |  |
| Agree  Disagree | 21  34 | 38.2  61.8 | 11  37 | 22.9  77.1 | 2.789 | .095 | .165 |
| When I talk about our fertility issues, my partner seems comforted by my comments |  |  |  |  |  |  |  |
| Agree  Disagree | 46  9 | 83.6  16.4 | 38  10 | 79.2  20.8 | .340 | .560 | .057 |
| My partner doesn’t understand the way fertility issues affect me |  |  |  |  |  |  |  |
| Agree  Disagree | 14  41 | 25.5  74.5 | 1  47 | 2.1  97.9 | 11.252 | .001 | .331 |
| My partner and I work well together handling questions about our infertility |  |  |  |  |  |  |  |
| Agree  Disagree | 51  4 | 92.7  7.3 | 47  1 | 2.1  97.9 | 1.494 | .222 | .120 |
| It bothers me that my partner reacts differently to our fertility issues |  |  |  |  |  |  |  |
| Agree  Disagree | 19  36 | 34.5  65.5 | 8  40 | 16.7  83.3 | 4.236 | .040 | .203 |
| My partner and I could talk more openly with each other about our fertility issues |  |  |  |  |  |  |  |
| Agree  Disagree | 35  20 | 63.6  36.4 | 32  16 | 66.7  33.3 | .104 | .748 | .032 |
| I couldn’t imagine us ever separating because of fertility issues |  |  |  |  |  |  |  |
| Agree  Disagree | 46  9 | 83.6  16.4 | 37  11 | 77.1  22.9 | .703 | .402 | .083 |
| When I try to talk about fertility issues, it seems to lead to an argument |  |  |  |  |  |  |  |
| Agree  Disagree | 5  50 | 9.1  90.9 | 0  48 | 0  100 | 4.586 | .032 | .211 |
| Because of infertility, I worry that my partner and I are drifting apart |  |  |  |  |  |  |  |
| Agree  Disagree | 3  52 | 5.5  94.5 | 5  43 | 10.4  89.6 | .881 | .348 | .092 |
